# Fear, Stress, Susceptibility, and Problematic Social Media Use Explain Motivation for COVID-19 Preventive Behaviors Among Patients With Stroke and Their Caregivers

**DOI:** 10.1177/00469580231225030

**Published:** 2024-02-05

**Authors:** Shikha Kukreti, Meng-Tsang Hsieh, Chieh-hsiu Liu, Jung-Sheng Chen, Yi-Jung Chen, Ming-Ta Hsieh, Chung-Ying Lin, Mark D. Griffiths

**Affiliations:** 1 Department of Nursing, College of Medicine, National Cheng Kung University, Tainan, Taiwan; 2 Stroke Center and Department of Neurology, Chi-Mei Medical Center, Tainan, Taiwan; 3 School of Medicine, College of Medicine, National Sun Yat-sen University, Kaohsiung, Taiwan; 4 Department of Family Medicine, Taoyuan General Hospital, Ministry of Health and Welfare, Taoyuan, Taiwan; 5 School of Medicine, National Tsing Hua University, Hsinchu, Taiwan; 6 Department of Medical Research, E-Da Hospital, I-Shou University, Kaohsiung, Taiwan; 7 Institute of Allied Health Sciences, College of Medicine, National Cheng Kung University, Taiwan; 8 Department of Family Medicine and Community Medicine, E-Da Hospital, I-Shou University, Kaohsiung, Taiwan; 9 School of Medicine, College of Medicine, I-Shou University, Kaohsiung, Taiwan; 10 Biostatistics Consulting Center, National Cheng Kung University Hospital, College of Medicine, National Cheng Kung University, Tainan, Taiwan; 11 Department of Occupational Therapy, College of Medicine, National Cheng Kung University, Tainan, Taiwan; 12 Faculty of Health and Life Sciences, INTI International University, Nilai, Malaysia; 13 International Gaming Research Unit, Psychology Department, Nottingham Trent University, Nottingham, UK

**Keywords:** caregiver, COVID-19, preventive behavior, stroke, vaccine acceptance

## Abstract

The COVID-19 pandemic presented significant challenges for individuals who experienced stroke and their caregivers. It is essential to understand the factors affecting preventive behavior in these populations. Therefore, the present study examined the factors that influenced COVID-19 preventive behavior and motivation for COVID-19 vaccine uptake among patients with stroke and their caregivers. A cross-sectional study comprising 191 participants (81 patients with stroke and 110 caregivers) was carried out. Participants completed a survey assessing fear of COVID-19, stress, perceived susceptibility, problematic social media use, preventive behaviors, and motivation for vaccine uptake. Statistical analyses included descriptive statistics, Pearson correlations, and multiple linear regressions. Motivation for COVID-19 vaccine uptake was significantly positively correlated with problematic social media use (r = 0.225, *P* = .002), perceived susceptibility (r = 0.197, *P* = .008), and fear of COVID-19 (r = 0.179, *P* = .015), but negatively correlated with stress (r = −0.189, *P* = .010). Caregivers, compared to patients, showed a lower level of preventive behavior (standardized coefficient = −0.23, *P* = .017). Furthermore, higher levels of fear were associated with increased preventive behavior (standardized coefficient = 0.22, *P* = .006), while greater stress correlated with lower preventive behavior (standardized coefficient = −0.38, *P* < .001). Among patients with stroke and their caregivers, motivation of COVID-19 vaccine uptake and preventive behaviors were influenced by factors such as fear, perceived susceptibility, social media use, and stress. By using strategies such as targeted education, support, and communication campaigns, healthcare providers and policymakers may be able to enhance the well-being of patients with stroke and their caregivers during future pandemics.

**What do we already know about this topic?**
Preventive behaviors and vaccine uptake are important factors for people not to be infected by the COVID-19.
**How does your research contribute to the field?**
The present study offers significant contributions to the field by enhancing understanding of preventive behaviors during crises, specifically for patients with stroke and their caregivers. It highlights the importance of a number of factors including fear, stress, and perceived susceptibility in influencing vaccine uptake and preventive behaviors. These insights are crucial for developing targeted interventions and effective communication strategies, especially in managing psychological stressors.
**What are your research’s implications toward theory, practice, or policy?**
Strategies to reduce stress and provide coping mechanisms for patients with stroke and caregivers of patients with stroke may enhance their ability to engage in preventive behaviors; additionally, healthcare providers should actively address and debunk misinformation on social media platforms to promote accurate and evidence-based information.

## Introduction

The COVID-19 pandemic posed significant challenges and concerns for individuals who experienced stroke and their caregivers. Caregivers often face increased stress and care responsibilities,^1^ while patients with stroke, especially those with underlying health conditions, are at a higher risk of developing severe illness if they contract COVID-19.^2^ However, there are measures that can be taken to mitigate these risks. One crucial step is getting vaccinated, a type of preventive behavior. Vaccination significantly reduces the chances of patients with stroke experiencing severe complications, hospitalization, and even death in the event of contracting COVID-19.^3^

By prioritizing vaccination, patients with stroke can safeguard their health and enhance their ability to recover. In addition to vaccination, practicing preventive measures such as mask-wearing, maintaining good hand hygiene, and practicing social distancing is essential for caregivers. These actions can reduce the risk of caregivers contracting and transmitting the virus to patients with stroke, who may already have compromised health or weakened immune systems. The Global Carer Well-being Index survey across 12 countries found that caregiving time markedly increased during the pandemic, with 20% of adults taking on caregiving roles for the first time.^4^ It is important to recognize that caregivers’ well-being is equally important because they play a crucial role in providing care and support. By adhering to preventive measures, caregivers can lower their risk of contracting COVID-19 and experiencing severe illness. This ensures their continued ability to provide care without disruptions. The COVID-19 pandemic posed unique challenges to patients with stroke and their caregivers.^5^ However, by prioritizing vaccination, practicing other preventive behaviors (in addition to vaccination), and prioritizing their own health, both patients with stroke and their caregivers can reduce their risk and enhance their well-being.

Fear can prompt individuals to take proactive measures to minimize their risks and regain a sense of control amidst uncertainty.^6,7^ COVID-19 vaccinations can be seen as a proactive measure to combat fear,^8^ giving caregivers a practical measure to protect themselves and their patients. Moreover, a previous study showed that fear of transmitting COVID-19 can motivate individuals to adopt preventive measures.^9^ However, it is essential to note that fear alone may not always lead to sustained behavior change. Greater levels of perceived susceptibility may also serve as important factor promoting COVID-19 preventive behavior^10^ and motivation to get vaccinated among adults living with chronic disease.^11^ Therefore, it is important to consider these factors within the specific context of caregivers of patients with stroke and patients with stroke to better understand their impact on behavior and vaccine motivation.

In a recent study, researchers found that caregivers of patients with stroke experienced higher care burdens, which was associated with lower acceptance of COVID-19 vaccines.^12^ Xie and colleagues reported a positive association between perceived information distortion regarding COVID-19 vaccination and social media use among participants with lower functional literacy.^13^ Problematic social media use has been defined as the excessive use of social media leading negative effects on the user’s professional, social and/or personal life.^14^ Moreover, it was reported by Ahorsu et al. that cyberchondria, fear of COVID-19 and perceptions of COVID-19 risk mediated indirect relationships between problematic social media use and COVID-19 vaccination.^15^

The role of problematic social media usage on care burden and COVID-19 vaccination among stroke caregivers has only been investigated in a few studies.^12^ Considering the limited literature and importance of this topic, it is evident that there is need to conduct further research on the factors that impact patients with stroke and their caregiver’s motivation to get vaccinated and engagement in preventive behaviors other than vaccination. Therefore, the present study examined factors (ie, fear of COVID-19, stress, perceived susceptibility, problematic social media use) that influence motivation for COVID-19 vaccine uptake and COVID-19 preventive behaviors (other than vaccination) among a sample of patients with stroke and caregivers. Understanding these factors among such populations is crucial for public health interventions and policy development. The present study aimed to provide new insights to the extant scientific knowledge base by investigating the following exploratory research questions (RQs): (i) how do specific factors (ie, fear of COVID-19, stress, perceived susceptibility, problematic social media use) influence COVID-19 preventive behaviors (other than vaccination) among patients with stroke and caregivers? and (ii) how do specific factors (ie, fear of COVID-19, stress, perceived susceptibility, problematic social media use) influence motivation for COVID-19 vaccine uptake among patients with stroke and caregivers?

## Methods and Materials

### Participants and Recruitment Procedure

The present study used a cross-sectional design. The study enrolled caregivers of patients with stroke in E-Da Hospital, and those patients with stroke who received consistent follow-up care in the outpatient department. To enhance the external validity of the study’s potential findings, both patients with stroke and their caregivers were included in the study. This approach was chosen to understand the influential factors for both groups. Primary caregivers were included in the study if they met the following criteria: (i) being aged over 20 years because the Taiwan Civil Law defines being aged 20 years as the legal age of an adult at the time of the study,^16^ (ii) caring for patients with stroke for more than 4 hours per day, and (iii) accompanying the patients during acute stroke phase (defined as the period occurring within 7 days after the onset of the stroke) and regular outpatient follow-ups (ie, the patients in the present study were regularly followed up at an interval of 1 to 3 months in the neurology outpatient clinic. According to the hospital’s regulations, follow-up components encompass a neurological examination, assessment of compliance with stroke prevention medications, evaluation of risk factors, and monitoring through cerebral and vascular imaging). Caregivers were excluded based on the following criteria: (i) not understanding or being able to complete the survey and (ii) having dementia or cognitive impairment, hearing loss, or psychiatric illnesses. More specifically, for caregivers with a history of dementia, cognitive impairment, or psychiatric illnesses, they were checked if they had relevant medical history in the present hospital. In cases where there was no such information, they were evaluated using in-person interview to assess their understanding of the questionnaire and the appropriateness of their responses during the initial interview by research assistants. If there were concerns related to any of the symptoms or disorders, a formal Mini-Mental State Examination (MMSE) was administered with confirmation of a diagnosis being sought through consultation with a neurologist or a psychiatrist. In cases where there was suspicion of hearing loss, the individuals’ auditory responses were assessed during the interview process. If concerns about hearing impairment arose, a calibrated finger rub auditory screening test or a formal hearing assessment was conducted.

Patients with stroke were included if they met the following criteria: (i) being aged over 20 years, (ii) having a diagnosis of stroke (ie, ischemic, hemorrhagic, and transient ischemic attack), and (iii) regularly receiving follow-ups in either outpatient or inpatient departments. The exclusion criteria were the same as those for caregivers. For screening eligibility of the participants, a brief neurological examination was performed, including the testing of higher cortical functions, and a MMSE and Clinical Dementia Rating for those suspected as having a cognitive impairment. Participants (either caregivers or patients with stroke) with hearing loss were excluded because they might not fully comprehend the questionnaire content. Additionally, participants who were illiterate and the examiners who were not proficient in sign language communication may have faced challenges in effectively conveying the questionnaire content.

The participants (including both caregivers and patients with stroke) completed the surveys during outpatient or inpatient follow-ups, and they were helped to complete the survey with verbal instructions by trained research assistants. The participants (either caregivers or patients with stroke) who were literate independently completed the paper-based questionnaires in a quiet room. For those who were illiterate, research assistants interviewed them and their verbal responses were recorded in the electronic questionnaire system. The research assistants in the present study had prior experience administering identical and related questionnaires in other research studies. They received training on the questionnaire content and its administration. The assistants were capable of providing explanations using language familiar to the participants, assisting in clarifying questionnaire items during the research, and conducting complete questionnaire surveys via interview for illiterate participants. The study was approved by the institutional review board (IRB) of E-Da Hospital (No. EMRP-110-079). All participants were informed about the study goals. Written informed consent was obtained from all participants prior to the study enrollment according to the Declaration of Helsinki. The study was conducted in accordance with the Declaration of Helsinki and the ethical guidelines for Ethical Guidelines for Medical and Health Research Involving Human Subjects in Taiwan.

The required sample size was calculated using the rule-of-thumb for multiple regression models (ie, the formula indicating 50 plus 10 participants per independent or controlled variable)^17^ Given that 9 independent and control variables were included in the regression models (please see ‘Statistical analysis’ section for details), the sample size calculation was: 50 + 10 × 9. Subsequently, the required sample size for the present study was 140.

### Measures

#### Motivation for vaccine uptake

Motivation for vaccine uptake was assessed using the Motors of COVID-19 Vaccine Acceptance Scale (MoVac-COVID19S).^18^ The MoVac-COVID19S comprises 12 items assessed on a 7-point Likert scale (ranging from “strongly disagree” to “strongly agree”).^19^ The MoVac-COVID19S item scores were unified in the same direction and summed (scores ranging between 12 and 84) with higher scores indicating greater motivation for COVID-19 vaccine uptake.^20,21^ An example item is “*Vaccination is a very effective way to protect me against COVID-19.*” The MoVac-COVID19S has been validated across different populations,^22^ including the Taiwanese population.^23^

#### Preventive behavior

COVID-19 preventive behaviors (excluding vaacination) was assessed using the Preventive COVID-19 Infection Behaviors Scale (PCIBS).^24^ The PCIBS contains 5 items rated on a five-point Likert scale (from “almost never” to “almost always”).^25^ The PCIBS item scores were unified in the same direction and averaged (scores ranging between 1 and 5) with higher scores indicating higher levels of COVID-19 preventive behaviors. An example item is “*How often do you avoid touching eyes, nose, and mouth?*” The PCIBS has been validated across different populations,^25^ including the Taiwanese population.^24^

#### Fear of COVID-19

Fear of COVID-19 was assessed using the Fear of COVID-19 Scale (FCV-19S). The FCV-19S contains 7 items rated on a five-point Likert scale (from “strongly disagree” to “strongly agree”).^26^ The FCV-19S item scores are summed with higher scores indicating greater fear of COVID-19.^24,27^ An example item is “*I am most afraid of COVID-19.*” The scale has been validated across different populations,^28,29^ including the Taiwanese population.^24^

#### Stress

Stress was assessed using a subscale of the Depression, Anxiety, and Stress Scale (DASS-21). The stress subscale of the DASS-21 contains 7 items rated on a 4-point Likert scale (from “did not apply to me at all” to “applied to me very much or most of the time”).^30^ The stress subscale item scores were summed with higher scores indicating greater general stress.^31^ An example item is “*I found it hard to wind down.*” The scale (including its stress subscale) has been validated across different populations,^36,37^ including the Taiwanese population.^32^

#### Perceived susceptibility to COVID-19

A self-reported single item (ie, “*How likely is it that you will be infected with COVID-19?*”) was used to assess perceived susceptibility (from “very unlikely” to “very likely”). Higher scores indicate greater perceived susceptibility to COVID-19.

#### Problematic social media use

Problematic social media use was assessed using the Bergen Social Media Addiction Scale (BSMAS). This scale comprises 6 questions, each answered on a 5-point Likert scale ranging from “very rarely” to “very often.”^33^ The total score of the BSMAS items is calculated by adding them together, with higher totals suggesting greater risk of problematic social media use.^34^ An example item is “*You spend a lot of time thinking about social media or planning how to use it.*” The BSMAS has been validated across different populations,^35-38^ including the Taiwanese population.^39,40^

#### Other covariates

Apart from the aforementioned measures, all participants completed a background information sheet to report their following information: age (reported in years), sex (male or female), marital status (married or other), number of years of education, and status (caregiver or patient).

### Statistical Analysis

The data were first analyzed using descriptive statistics to separately summarize the patients’ and caregivers’ demographics and measure scores. In order to determine bivariate correlations between the studied variables (including age, sex, number of years of education, marital status, status [patient or caregiver], fear of COVID-19, stress, perceived susceptibility, problematic social media use, preventive behaviors, and motivation for COVID-19 vaccine uptake), Pearson correlations were used. Lastly, two multiple linear regressions were constructed using the same set of independent variables and confounders but different dependent variables. The independent variables included fear of COVID-19, stress, perceived susceptibility, and problematic social media use. The confounders included age, sex (female as reference group), marital status (married as reference group), and status (patient as reference group). For the first regression model, preventive behaviors was the dependent variable. For the second regression model, motivation for COVID-19 vaccine uptake was the dependent variable. All the statistical analyses were performed using IBM SPSS 20.0 (IBM Incorp.: Armonk, NY).

## Results

Among the 191 participants, 81 were patients with stroke (mean [SD] age = 64.71 years [SD = 7.78]; 71.60% males) and 110 were caregivers of patients with stroke (mean age = 60.58 years [SD = 7.37]; 30.91% males). Both subsamples (ie, patients and caregivers) had a mean number of years in education above 9 years and were mostly married (87.65% among patients and 90.00% in caregivers). Table 1 additionally reports the scores for fear of COVID-19, stress, perceived susceptibility, problematic social media use, preventive behaviors, and motivation for COVID-19 vaccine uptake for the two subsamples separately.

**Table 1.** Caregiver Characteristics (N = 191).

Variables	Patients (n = 81)	Caregiver (n = 110)
Age (in years); mean (SD)	64.71 (7.78)	60.58 (7.37)
Sex; n (%)		
Male	58 (71.60)	34 (30.91)
Female	23 (28.40)	76 (69.09)
Years of education; mean (SD)	9.94 (7.74)	9.64 (3.84)
Marital status; n (%)		
Married	71 (87.65)	99 (90.00)
Other/Missing	10 (12.35)	11 (10.00)
Fear of COVID-19; mean (SD)	8.83 (4.56)	8.42 (3.20)
Stress; mean (SD)	1.01 (2.17)	1.07 (2.06)
Perceived susceptibility; mean (SD)	1.53 (0.93)	1.75 (0.98)
Problematic social media use; mean (SD)	6.67 (1.36)	7.26 (1.94)
Preventive behaviors; mean (SD)	4.55 (0.26)	4.28 (0.71)
Motivation of COVID-19 vaccine uptake; mean (SD)	61.32 (8.70)	60.08 (10.48)

*Note*. Possible score range: 7 to 35 for fear of COVID-19; 0 to 21 for stress; 1 to 5 for perceived susceptibility; 6 to 30 for problematic social media use; 1 to 5 for preventive behaviors; and 12 to 84 for motivation of COVID-19 vaccine uptake.

Table 2 reports the correlation coefficients between the studied variables. Motivation for COVID-19 vaccine uptake was significantly associated with problematic social media use (r = .225; *P* = .002), perceived susceptibility (r = .197; *P* = .008), stress (r = −.189; *P* = .010), and fear of COVID-19 (r = .179; *P* = .015). Preventive behaviors was significantly associated with stress (r = −.310; *P* < .001) and marginally associated with fear of COVID-19 (r = .136; *P* = .066).

**Table 2.** Correlation Matrix Using Pearson Correlations for Studied Variables.

	r (*P*-value)										
Variables	1.	2.	3.	4.	5.	6.	7.	8.	9.	10.	11.
1. Age	—										
2. Sex	−.058 (.427)	—									
3. Education	−.233 (.002)	−.229 (.002)	—								
4. Marital status	−.094 (.195)	−.164 (.024)	.009 (.907)	—							
5. Patient	−.263 (<.001)	.403 (<.001)	−.025 (.736)	−.037 (.611)	—						
6. Fear	.057 (.439)	.142 (.054)	−.083 (.274)	−.096 (.195)	−.053 (.475)	—					
7. Stress	−.099 (.180)	.165 (.025)	−.135 (.072)	−.007 (.921)	.013 (.860)	.305 (<.001)	—				
8. PS	.074 (.327)	.160 (.032)	−.085 (.265)	.019 (.799)	.115 (.124)	.420 (<.001)	.048 (.522)	—			
9. PSMU	−.259 (<.001)	−.074 (.314)	.217 (.004)	.172 (.019)	.169 (.021)	.121 (.107)	−.065 (.384)	.036 (.642)	—		
10. Behaviors	.063 (.395)	−.193 (.009)	.046 (.547)	−.049 (.512)	−.240 (.001)	.136 (.066)	−.310 (<.001)	.024 (.754)	.087 (.248)	—	
11. Motivation	.128 (.084)	−.053 (.478)	.114 (.131)	.069 (.350)	−.064 (.391)	.179 (.015)	−.189 (.010)	.197 (.008)	.225 (.002)	.349 (<.001)	—

Education = years of education; patient = patient or caregiver; fear = fear of COVID-19; PS = perceived susceptibility; PSMU = problematic social media use; behaviors = preventive behaviors; motivation = motivation of COVID-19 vaccine uptake.

The first multiple linear regression showed that caregivers as compared to patients had a lower level of preventive behavior (standardized coefficient (SC) = −0.23; *P* = .017), greater fear was associated with a higher level of preventive behavior (SC = 0.22; *P* = .006), and greater stress was associated with a lower level of preventive behavior (SC = −0.38; *P* < .001) (Table 3). The second multiple linear regression showed that a higher level of fear of COVID-19 (SC = 0.20; *P* = .014), perceived susceptibility (SC = 0.17; *P* = .033), and problematic social media use (SC = 0.16; *P* = .036) were associated with a higher motivation for COVID-19 vaccine uptake. Stress (SC = −0.18; *P* = .015) was associated with a lower motivation for COVID-19 vaccine uptake (Table 3).

**Table 3.** Linear Regression Models Explaining Preventive Behaviors and Motivation of Vaccine Uptake.

Dependent variable: preventive behaviors (R^2^ = 0.26; Adj. R^2^ = 0.22; entire model *P*-value < .001)	Unstand. Coeff. (SE)	Stand. Coeff.	*P*-value
Age (year)	0.0003 (0.01)	0.004	.960
Sex (Ref: female)	0.12 (0.09)	0.11	.198
Marital status (Ref: married)	−0.11 (0.13)	−0.06	.423
Educational year	0.00 (0.01)	−0.03	.687
Patient or caregiver (Ref: patient)	−0.23 (0.10)	−0.20*	.017
Fear of COVID-19	0.04 (0.01)	0.22*	.006
Stress	−0.11 (0.02)	−0.38*	<.001
Perceived susceptibility	−0.01 (0.05)	−0.01	.860
Problematic social media use	0.04 (0.03)	0.10	.168
Dependent variable: motivation of vaccine uptake (R^2^ = 0.21; Adj. R^2^ = 0.17; entire model *P*-value = <.001)	Unstand. Coeff. (SE)	Stand. Coeff.	*P*-value
Age (year)	0.17 (0.10)	0.14	.076
Sex (Ref: female)	0.43 (1.65)	0.02	.797
Marital status (Ref: married)	3.76 (2.31)	0.12	.107
Educational year	0.21 (0.12)	0.13	.093
Patient or caregiver (Ref: patient)	−1.06 (1.67)	−0.05	.526
Fear of COVID-19	0.57 (0.23)	0.20*	.014
Stress	−0.85 (0.35)	−0.18*	.015
Perceived susceptibility	1.79 (0.83)	0.17*	.033
Problematic social media use	0.98 (0.46)	0.16*	.036

Unstand. Coeff. = unstandardized coefficient; SE = standard error; Stand. Coeff. = standardized coefficient.

* *P* < .05.

## Discussion

The findings of the present study provide valuable insights into the factors that influence COVID-19 preventive behaviors (excluding vaccination) and motivation for COVID-19 vaccine uptake among patients with stroke and caregivers of patients with stroke. The study findings showed a positive association of fear of COVID-19 with both motivation for vaccine uptake and preventive behavior. Fear can serve as a powerful motivator, prompting individuals to take proactive measures to minimize their risks and regain a sense of control amidst uncertainty.

In the context of stroke (either patients or caregivers), fear of transmitting COVID-19 to vulnerable patients can be a strong motivator for caregivers to adopt preventive measures and prioritize vaccination. The present study’s findings concur with previous research highlighting the role fear of COVID-19 plays in motivating individuals to engage in protective behaviors^41^ and seek vaccination.^8^ However, the present study advances knowledge by providing evidence regarding patients with stroke and their caregivers.

The present study also found a negative association between stress and preventive behaviors. This suggests that higher levels of stress may hinder caregivers’ ability to engage in preventive behaviors. It is understandable that caregivers of patients with stroke may experience high levels of stress given the increased responsibilities and challenges they face. Addressing stress and providing support for caregivers to manage their stress levels may be crucial in promoting and sustaining preventive behaviors.

It was found that problematic social media use was positively associated with motivation for vaccine uptake. Social media can play a significant role in shaping attitudes and behaviors.^42^ In the context of COVID-19, social media platforms have been both a source of information and misinformation. Caregivers who engage in problematic social media use may be more exposed to misleading or inaccurate information regarding vaccines, potentially affecting their motivation to get vaccinated. It is important for healthcare providers and public health authorities to counter misinformation with accurate information and provide guidance on reliable sources of information to ensure that caregivers can make informed decisions.

Perceived susceptibility was another factor in the present study positively associated with motivation for vaccine uptake. This suggests that individuals who perceive themselves to be at higher risk of contracting COVID-19 may be more motivated to get vaccinated. This finding is consistent with previous research that has shown the importance of perceived susceptibility in motivating individuals to engage in protective behaviors.^43^ Healthcare providers can play a role in educating patients with stroke and their caregivers about the increased risk associated with underlying health conditions and the benefits of vaccination in reducing the severity of illness.

Overall, the findings of the present study highlights the importance of addressing fear, stress, social media use, and perceived susceptibility in promoting COVID-19 preventive behaviors and vaccine uptake among patients with stroke and their caregivers. Public health interventions and policies should focus on addressing these factors through targeted education, support, and communication campaigns. Strategies to reduce stress and provide coping mechanisms for caregivers may also enhance their ability to engage in preventive behaviors. Additionally, healthcare providers should actively address and debunk misinformation on social media platforms to promote accurate and evidence-based information.

There are some limitations to the present study that should be acknowledged. First, it used a cross-sectional design, which limits the ability to establish causal relationships. Future longitudinal studies are needed to examine these relationships over time. Second, the study sample was limited to caregivers and patients with stroke from a single hospital, which means the sample was small and may limit the generalizability of the findings. Including a larger and more diverse sample from multiple healthcare settings would strengthen the study’s findings. Third, the study relied on self-report measures, which may be subject to response bias. Future studies could consider using objective measures (such as physiological or behavioral assessment) or a combination of self-report and objective measures to enhance the validity of the findings.

## Conclusion

The present study emphasizes the importance of addressing fear, stress, social media use, and perceived susceptibility in promoting COVID-19 preventive behaviors and vaccine uptake among patients with stroke and caregivers of patients with stroke. By understanding these factors and tailoring interventions accordingly, healthcare providers and policymakers can effectively support patients with stroke and their caregivers in navigating the challenges posed by the COVID-19 pandemic (and those in the future) and ensure their well-being. A comprehensive and holistic approach that addresses the unique needs and concerns of caregivers and patients with stroke are likely to contribute to better outcomes and reduce the burden on healthcare systems.
